# Lysolipids regulate raft size distribution

**DOI:** 10.3389/fmolb.2022.1021321

**Published:** 2022-10-05

**Authors:** Vladimir D. Krasnobaev, Timur R. Galimzyanov, Sergey A. Akimov, Oleg V. Batishchev

**Affiliations:** ^1^ Frumkin Institute of Physical Chemistry and Electrochemistry, Russian Academy of Sciences, Moscow, Russia; ^2^ Moscow Institute of Physics and Technology (National Research University), Dolgoprudny, Russia

**Keywords:** lipid rafts∗, neurodegenerative diseases, Alzheimer disease, lysolipids, cholesterol, line-active components, atomic force microscopy, theory of elasticity

## Abstract

The lipid matrix of cellular membranes, directly and indirectly, regulates many vital functions of the cell. The diversity of lipids in membranes leads to the formation of ordered domains called rafts, which play a crucial role in signal transduction, protein sorting and other cellular processes. Rafts are believed to impact the development of different neurodegenerative diseases, such as Alzheimer’s, Parkinson’s, Huntington’s ones, amyotrophic lateral sclerosis, some types of cancer, etc. These diseases correlate with the change in the membrane lipid composition resulting from an oxidative stress, age-related processes, dysfunction of proteins, and many others. In particular, a lot of studies report a significant rise in the level of lysolipids. Physicochemical properties of rafts are determined by membrane composition, in particular, by the content of lysolipids. Lysolipids may thus regulate raft-involving processes. However, the exact mechanism of such regulation is unknown. Although studying rafts *in vivo* still seems to be rather complicated, liquid-ordered domains are well observed in model systems. In the present study, we used atomic force microscopy (AFM) to examine how lysophospholipids influence the liquid-ordered domains in model ternary membranes. We demonstrated that even a small amount of lysolipids in a membrane significantly impacts domain size depending on the saturation of the lysolipid hydrocarbon tails and the amount of cholesterol. The mixture with the bigger relative fraction of cholesterol was more susceptible to the action of lysolipids. This data helped us to generalize our previous theoretical model of the domain size regulation by lipids with particular molecular shape expanding it to the case of lysolipids and dioleoylglycerol.

## 1 Introduction

The lipid matrix of cellular membranes contains thousands of different lipids. The need for this diversity is still a puzzle. Moreover, different biochemical reactions occurring outside and inside the cell may change the lipid composition, e.g., in the case of lipid peroxidation at inflammation and oxidative stress, or aberrant lipid metabolism. These alterations in the amount of particular lipid species are found to be correlated with the development of severe diseases. The lipid composition of the brain tissue is related to the development of neurodegenerative disorders, such as Alzheimer’s (AD), Parkinson’s (PD), Huntington’s (HD) ones, and amyotrophic lateral sclerosis (ALS). It is demonstrated that phospholipids are modified in brains affected by AD (SoOderberg et al., 1992). In particular, compositional changes in cellular lipid membranes occur at early stages of sporadic AD (Fabelo et al., 2014) and in different types of cancer (Szlasa et al., 2020). The lipid composition changes as well in the frontal and visual cortex in case of PD (Cheng et al., 2011; Wood et al., 2018; Fanning et al., 2020; Stok and Ashkenazi, 2020), and spinal cord in ALS (Chaves-Filho et al., 2019; Dodge et al., 2020). There are several studies reporting alternated levels of phospholipids circulating in a blood plasma as a possible biomarker of AD (Goodenowe et al., 2007; González-Domínguez et al., 2014; Mapstone et al., 2014). Among them, lysophospholipids attract great attention (Dorninger et al., 2018). The change of cellular, and, consequently, blood level of lysolipids could be the result of oxidative stress and inflammations related to the development of neurodegenerative diseases (Girotti and Kriska, 2004; Scholte et al., 2019). Moreover, it is well established that the high level of lysolipids produces pores in the lipid matrix of cellular membranes (Chernomordik et al., 1995; Mills and Needham, 2005). However, it is still unclear what could be the action of the small amount of lysolipids in cells, which, normally, possess various mechanisms to sustain their membrane integrity by the dynamic change in lipid composition, e.g., by the change in the cholesterol content (Khandelia et al., 2014).

It is a common knowledge that cellular membranes should be laterally fluid to maintain the normal cell homeostasis and functioning of the proteins (Quinn, 1981). Despite that, some lipids with high phase transition temperature may organize into liquid-ordered lipid domains, also called rafts, which are assumed to be important participants in different cellular processes, mainly signal transduction and membrane trafficking (Simons and Ikonen, 1997). Lipid rafts play crucial roles in many cell processes, such as apoptosis (Deans et al., 2002), endocytosis (Parton and Richards, 2003), exocytosis (Salaün et al., 2004) and even development of the viral infection (Suomalainen, 2002; Molotkovsky et al., 2018). The role of lipid rafts in the development of AD can be hardly exaggerated, as they are the platform for the processing of the Amyloid Precursor Protein with β- and γ-secretase (Lee et al., 1998; Riddell et al., 2001; Ehehalt et al., 2003; Molander-Melin et al., 2005; Grimm et al., 2008; Rushworth and Hooper, 2010; Hicks et al., 2012) in cholesterol-dependent manner (Ehehalt et al., 2003). Also, lipid rafts are also important for some other neurodegenerative diseases, such as PD and ALS (Schengrund, 2010). In a course of lateral phase separation, liquid-ordered domains can form spontaneously and exist without any protein in model lipid membranes containing saturated lipids, unsaturated lipids, and sterols (Dietrich et al., 2001). This indicates that lipid domains might influence the function of a protein, which preferentially partition into rafts or to their vicinity. The physical properties of these domains, ways and consequences of influencing them, and even their existence in living cells at physiological temperatures are still the matter of debates (Munro, 2003). Nevertheless, we know that model liquid-ordered domains are thicker than the rest of a membrane (Rinia et al., 2001; García-Sáez et al., 2007; Heftberger et al., 2015), have a bilayer structure (Kiessling et al., 2009; Saitov et al., 2020), and they are in a more ordered phase state than a surrounding membrane (Heberle and Feigenson, 2011). These raft-in and raft-out bulk phases are usually referred to as liquid-ordered (L_o_) and -disordered (L_d_) phases, respectively. The mismatch of the bilayer thickness between L_d_ and L_o_ phases is smoothed by the elastic deformation of lipids along the domain boundary (Kuzmin et al., 2005). In this way, formation of a raft boundary requires an energy spent on the deformations along with the excess energy arising from the lipid concentration difference in L_o_ and L_d_ phases. The total excess interphase energy related to the unit length of the boundary is referred to as the line tension. Thus, even a raft itself is not laterally homogeneous, and some molecules, called lineactants (line-active components) (Trabelsi et al., 2008) may prefer or avoid its boundary more than the bulk phase. Recently, we have demonstrated theoretically that molecules inducing non-zero spontaneous curvature (SC) in lipid monolayers influence the deformational energy at the domain boundary, and, consequently, the line tension and the domain size (Pinigin et al., 2020). These molecules accumulate in a narrow region near the domain boundary, that allows them to substantially alter the elastic energy of the boundary even when the average concentration of the line-active component is very low (fractions of mole percent). In case of lipids, the spontaneous curvature of the monolayer results from the difference in cross-sectional areas of polar and hydrophobic parts of the lipid molecule. One example of the molecules with the significant positive spontaneous curvature is glycolipids, which possess a huge polar carbohydrate part. We have experimentally proved the theoretical prediction on line activity for ganglioside GM1 and discovered that even a tiny amount of this lipid significantly changes the raft size distribution depending on the amount of cholesterol in the membrane (Galimzyanov et al., 2017). Gangliosides are important regulatory lipids, and change in their level is associated with neurodegenerative disorders, especially AD, and tumor-induced apoptosis of T cells (Das et al., 2008; Yanagisawa, 2015; Magistretti et al., 2019).

Lysophospholipids have only one hydrocarbon tail in each molecule instead of two. Hence, they have a higher positive spontaneous curvature than general phospholipids, and should impact the line tension of rafts. From the physical point of view, we should not observe a principal difference between ganglioside and lysolipid action on the raft size distribution. There is a little evidence for lysophospholipids directly influencing domain formation (Ma et al., 2010; Knuplez et al., 2020), but the full mechanism of it is still unclear.

In the present study we tried to clarify whether domain size in model membranes is influenced by lysolipids in the same way as by GM1, and how perceptible the effect is. We utilized atomic force microscopy to experimentally show the changes in the size and height of L_o_ domains. Also, we used previously described theoretical model based on the theory of elasticity of liquid crystals to show the influence of lipid molecules with non-cylindrical shape on the line tension of raft boundaries. According to our hypothesis, as lysolipids form a monolayer with a positive spontaneous curvature, they can exhibit line activity, and significantly impact structure of the domain boundaries and raft sizes. Thus, they can regulate raft-associated processes. For the control, we made similar experiments with dioleoylglycerol possessing highly negative spontaneous curvature to demonstrate the generality of the approach.

## 2 Materials and methods

### 2.1 Materials

1,2-dioleoyl-sn-glycero-3-phosphocholine (DOPC), egg sphingomyelin (eSM), 1-oleoyl-2-hydroxy-sn-glycero-3-phosphatidylcholine (O-lysoPC), 1-palmytoyl-2-hydroxy-sn-glycero-3-phosphatidylcholine (P-lysoPC), 1,2-dioleoyl-sn-glycerol (DOG), and cholesterol (Chol) were purchased from Avanti Polar Lipids (Alabaster, AL, United States). Methanol (>99.0%) and chloroform (>99.0%) were purchased from Sigma-Aldrich (St. Louis, MO, United States). All chemicals were used without further purification. In experiments, we used deionized water with a resistivity of 18.2 MΩ cm. For further preparation, eSM was dissolved in Chloroform: Methanol (9:1, v:v) to the concentration of 5 g/L, other lipids were dissolved in chloroform to the final concentration of 10 g/L.

### 2.2 Atomic force microscopy

The lipid solution with a given molar ratio of the components was rotary evaporated in a glass vial under the low vacuum (200 mbar) at 36°C for 10 min and then stayed under the high vacuum (50 mbar) for another 30 min. The dried lipid film was resuspended in deionized water to the final lipid concentration of 0.5 g/L. The vial with the suspension was then sonicated for 40 min to form small vesicles. During the sonication, the vial was kept at 50°C. Vesicle suspensions were used immediately or stored at 4°C and used next day, re-sonicated and re-heated.

AFM experiments were performed using the Multimode Nanoscope V (Bruker, Billerica, MA, United States) setup equipped with the electrochemical fluid cell. A sample of 100 μl of the vesicle suspension pre-heated to 55°C was deposited on a freshly cleaved mica and incubated for 5 min. The vesicles ruptured and formed a lipid film at the mica surface. The obtained lipid film was then rinsed extensively with Milli-Q water of room temperature for three times to remove lipid multilayers, and put into a fluid cell for further AFM imaging. The sample was placed into the cell with 50 μl of Milli-Q water. We used Silicon-tip on Nitride Lever (SNL-10) cantilevers with a nominal spring constant of 0.06 N/m and the tip radius of approximately 2 nm (Bruker, Billerica, MA, United States). The images were scanned at dimensions of 3 μm^2^ × 3 μm^2^ and 10 μm^2^ × 10 μm^2^, and processed using WSxM software (Horcas et al., 2007). To analyze the size of individual domains we utilized the procedure described in the ref (Galimzyanov et al., 2017). In brief, because the shape of domains was not perfectly circular, meaning merging of L_o_ domains over time, the area of individual domains may change in time. Their boundary represents a combination of circular arcs, which reflect the size of initial domains and might be interpolated by corresponding circles. Therefore, we overlapped circles of different diameter over domains of liquid-ordered phase near its boundary to find the best match, and the average diameter of such circles was set as the raft diameter for given lipid composition. At least 100 domains from 3–5 independent AFM images were analyzed. Height of the domains was set as the difference between centers of the height distribution peaks for the L_d_ surrounding membrane and L_o_ domains using NanoScope Analysis software (Bruker, Billerica, MA, United States). At least 100 domains from 3–5 independent AFM images were analyzed for each case.

### 2.3 Theoretical analysis

**FIGURE 1:**
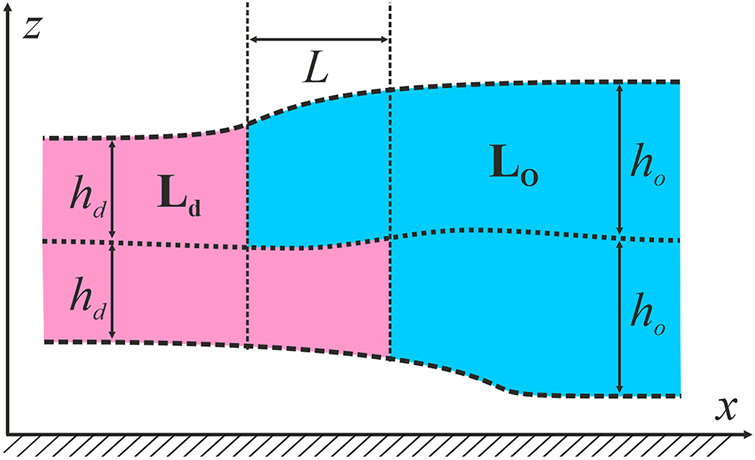
Schematic cross-section of the raft and the surrounding membrane by the plane perpendicular to the phase boundary. The raft is shown in blue; the surrounding membrane is pink. A Cartesian coordinate system is introduced: *Ox* axis is perpendicular to the raft boundary, *Oy* axis is parallel to the boundary, *Oz* axis is perpendicular to the membrane plane.

We considered the specific energy of ordered domains boundary (line tension) as the main factor governing domain size in the raft ensemble (Galimzyanov et al., 2017). From AFM (Rinia et al., 2001; García-Sáez et al., 2007) and X-Ray diffraction experiments (Heftberger et al., 2015), it is known that the bilayer of the L_o_ phase is 0.5–1.5 nm thicker than the L_d_ surrounding membrane. If the phases are homogeneous up to the boundary, the jump of bilayer thickness would result in huge line tension, which is about two orders of magnitude larger than the experimentally determined one (Baumgart et al., 2003). It is usually assumed that the thickness mismatch is compensated by elastic deformations of the membrane. The energy of deformations contributes to the line tension of the domain boundary; this contribution is frequently considered as the major one (Kuzmin et al., 2005; Galimzyanov et al., 2015, 2017; Staneva et al., 2016; Pinigin et al., 2020). We considered the bilayer lipid membrane as a continuous liquid-crystalline elastic medium. Calculations of the line tension were made in the framework of the model thoroughly described in our previous works (Galimzyanov et al., 2015, 2016, 2017; Staneva et al., 2016; Pinigin et al., 2020), therefore here we provide only main points of the approach. The smallest domains found in the experiments had a diameter of about 30 nm (Saitov et al., 2020) that is much larger than the characteristic length of elastic deformations, which are about several nanometers (Galimzyanov et al., 2015, 2016). Therefore, we considered the domain boundary as a straight line and assumed that locally the system possessed translational symmetry along the boundary that allowed treating it as effectively one-dimensional. As we have shown in the ref (Galimzyanov et al., 2011, Galimzyanov et al., 2015), the line tension of the boundary of the L_o_ domain is minimal when boundaries of two opposing monolayer domains are not completely in register, but relatively shifted by some equilibrium distance of about 2–4 nm. The distance between the boundaries of monolayer domains in the opposed monolayers is denoted as *L*. The thickness of the undeformed L_o_ monolayer is denoted as *h*_*o*_, the thickness of the undeformed L_d_ monolayer is denoted as *h*_*d*_ (Figure 1).

In the framework of the elastic approach, we considered lysolipids as membrane components with positive spontaneous curvature. Lysolipids could laterally distribute into L_o_ and L_d_ phases, as well as into the intermediate region between them. We used the approach described in (Galimzyanov et al., 2017), according to which added species distributed into the domain boundary in the form of the stripe parallel to the raft boundary. We used two types of lysolipids: P-lysoPC and O-lysoPC, the first one had a saturated acyl chain (palmitoyl), the second lipid had unsaturated lipid tail (oleoyl). To additionally prove the validity of the model, we also made calculations for the lipid with negative spontaneous curvature, DOG. Following the predictions of the elastic model, this lipid should also influence the line tension of the raft boundary.

To model an impact of lysolipids and DOG on the line tension of the domain boundary, we accounted for the effect of these components on the spontaneous curvature of the lipid monolayer. We used the standard approach, where the spontaneous curvature of the lipid mixture is considered to be equal to the weighted average on concentrations (mole fractions) of spontaneous curvatures of components (Kollmitzer et al., 2013). The monolayer spontaneous curvature *J*_*o,d*_ of bulk ordered and disordered phases are proportional to the concentration of lysolipid (or DOG) *x*_*o,d*_ in these phases:

$$J_{o,d}=J_{0;o,d}\left(1-x_{o,d}\right)+J_{L}x_{o,d},$$ (1)

where *J*_*L*_ is the spontaneous curvature of the pure lysolipid (or DOG) monolayer, *J*_0;*o,d*_ is the spontaneous curvature of monolayers of L_o_ and L_d_ phases containing no lysolipids (or DOG). The spontaneous curvature of the stripe containing lysolipid (or DOG) is determined similarly:

$$J_{st}=J_{0,st}\left(1-x_{st}\right)+J_{L}x_{st},$$ (2)

where *x*_*st*_ is the lysolipid (or DOG) concentration in the stripe; *J*_0,*st*_ is the default spontaneous curvature of stripe monolayers. Lysolipid (or DOG) concentration in the bulk phase and in the stripe in each monolayer should obey the condition of matter conservation:

$$S_{o}x_{o}+S_{d}x_{d}+L_{st}d_{st}x_{st}=S_{0}x_{L}$$, (3)

where *S*_*o*_ and *S*_*d*_ are the areas of the ordered and disordered phase, *L*_*st*_ is the length of the boundary of the raft phase, *d*_*st*_ is the width of the stripe enriched by lysolipid (or DOG), *S*_0_ is the total area of the lipid monolayer surface, *x*_*L*_ is the total concentration of lysolipid (or DOG) in the system.

We minimized the free energy of the system with respect to lateral distribution of deformations as well as the location, width, and composition of the stripe containing lysolipids (or DOG) under the condition of Eq. 3. Dividing the energy of the boundary *W* by its length, we obtained the line tension of the raft boundary, *γ*.

## 3 Results

### 3.1 Atomic force microscopy experiments

**FIGURE 2:**
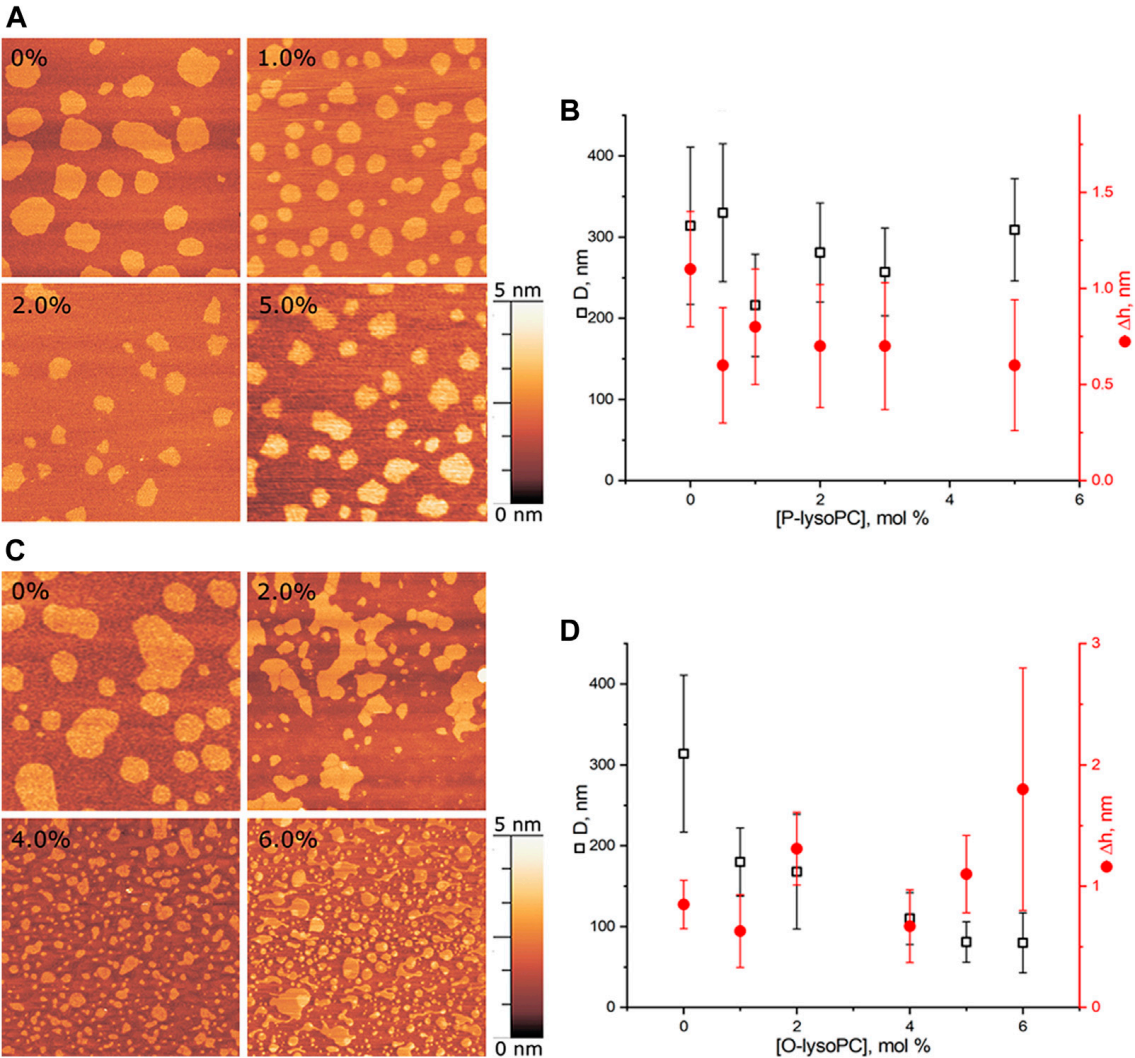
Lysolipids in the eSM/DOPC/Chol = 2:2:1 membrane. **(A)** AFM images of the membrane with P-lysoPC. **(B)** Dependence of the average diameter of rafts on the molar fraction of P-lysoPC in the membrane. **(C)** AFM images of the membrane with O-lysoPC. **(D)** Dependence of the average diameter of rafts (black) and their height above the surrounding membrane (red) on the molar fraction of O-lysoPC in the membrane. Size of each AFM image is 3 μm × 3 μm. Amount of lysolipids in the membrane in mol% is indicated in each image.

**FIGURE 3:**
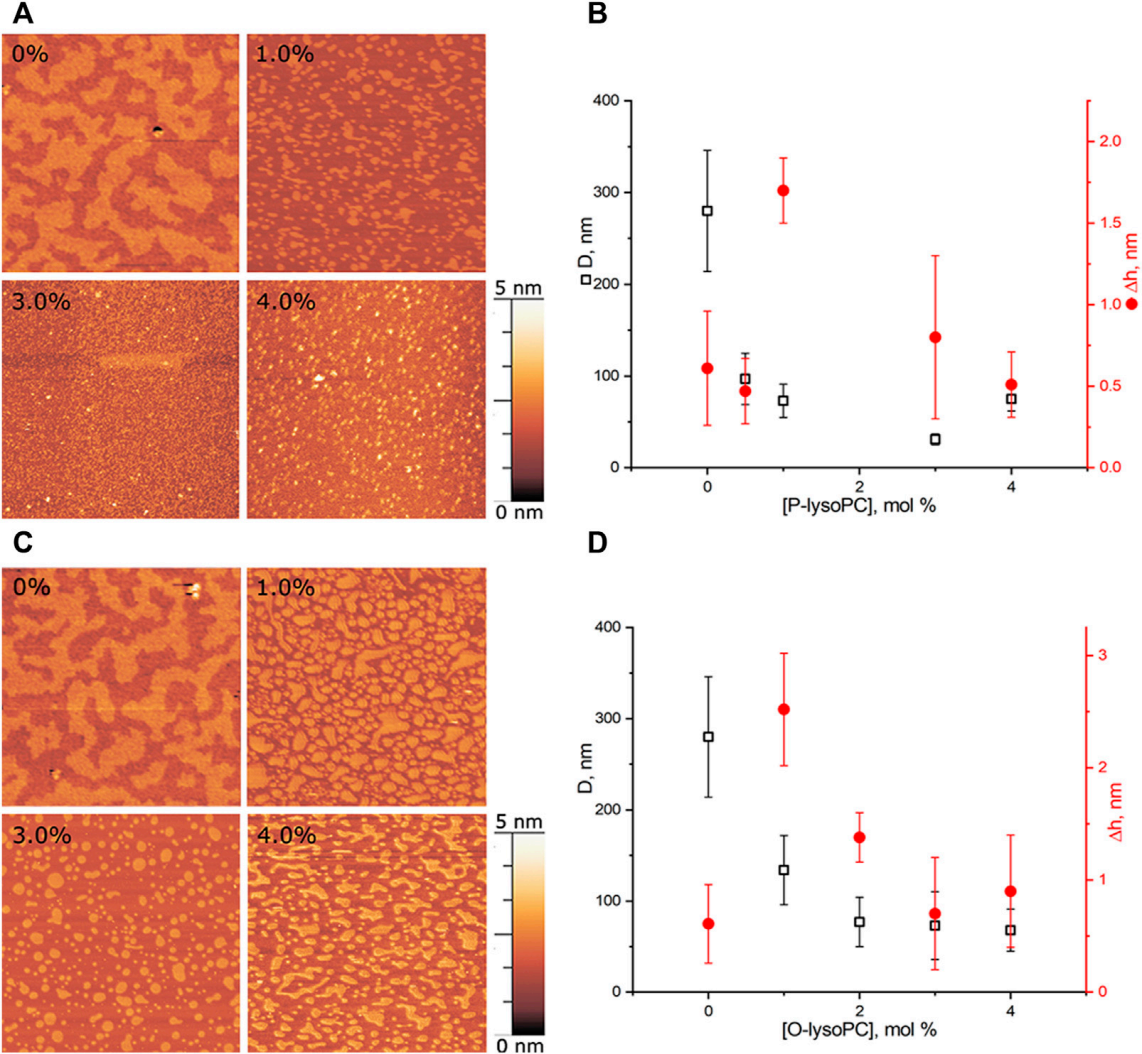
Lysolipids in the eSM/DOPC/Chol = 1:1:1 membrane **(A)** AFM images of the membrane with P-lysoPC. **(B)** Dependence of the average diameter of rafts on the molar fraction of P-lysoPC in the membrane. **(C)** AFM images of the membrane with O-lysoPC. **(D)** Dependence of the average diameter of rafts (black) and their height above the surrounding membrane (red) on the molar fraction of O-lysoPC in the membrane. Size of each AFM image is 3 μm × 3 μm. Amount of lysolipids in the membrane in mol% is indicated in each image.

We performed experiments in three-component bilayers of eSM/DOPC/Chol with molar ratio of components fixed at 2:2:1 or at 1:1:1 with different mole fractions of O-lysoPC, P-lysoPC, and DOG. Under the described conditions the 2:2:1 bilayer without lysolipids or DOG gave a clear picture of nearly circular rafts with the average diameter of (310 ± 100) nm and the height of around 1 nm (Figure 2) over the surrounding membrane that was in a good agreement with typical values for such systems (Khadka et al., 2015). The 1:1:1 membrane had domains with the average diameter of (280 ± 70) nm and the same height difference, Δh, with the surrounding membrane of 1 nm (Figure 3) that is similar to our previous observations (Galimzyanov et al., 2017). In this membrane the domains were less circular than in the 2:2:1 system, and mostly clustered, but still recognizable as separate. Rarely, domains were not uniform, having small holes inside them.

#### 3.1.1 Lysolipids in eSM/DOPC/Chol = 2:2:1 membrane

Adding P-lysoPC to the 2:2:1 membrane slightly reduced the average raft diameter, from the initial (310 ± 100) nm to the minimal (220 ± 60) nm at 1 mol% of P-lysoPC. However, the statistical accuracy forbids us to make any strong conclusions about that, and, in general, we can conclude about no change in the domain size in this system with increasing fraction of P-lysoPC (Figures 2A,B). Δh values were in a range of 0.25–1.5 nm without any monotonic dependence on the amount of P-lysoPC in the membrane.

For O-lysoPC we observed a different picture (Figure 2C). The addition of O-lysoPC made rafts less circular and monotonically reduced their average diameter down to (80 ± 40) nm at 6 mol% of O-lysoPC (Figure 2D). Δh values had a wider dispersion, from 0.2 to 2.5 nm, but with the same average value of about 1 nm.

#### 3.1.2 Lysolipids in eSM/DOPC/Chol = 1:1:1 membrane

In this system, presence of 0.5 mol% of P-lysoPC in the membrane reduced the average domain diameter three-fold at 1 mol% of P-lysoPC, from initial (280 ± 70) nm to (100 ± 30) nm (Figure 3A). The further increase of P-lysoPC concentration decreased the domain size gradually to 30 nm at 3 mol% of P-lysoPC (Figure 3B). However, at the concentration of 4.0 mol% domains became bigger again and we observed the same average domain size as in the case of 1.0 mol% of P-lysoPC.

The similar non-monotonic dependence of the average domain size on the molar fraction of the line-active component we observed for GM1 in the same (1:1:1 eSM/DOPC/Chol) lipid system (Galimzyanov et al., 2017). Notably, there was no such growth in the size of domains in the 2:2:1 eSM/DOPC/Chol mixture neither for studied lysolipids (Figure 2), nor for GM1 (Galimzyanov et al., 2017): the domain size decreased monotonically as the mole fraction of the line-active component increased. The mixtures eSM/DOPC/Chol = 1:1:1 and eSM/DOPC/Chol = 2:2:1 differ only by the amount of cholesterol and this fact indicates that lysolipid-cholesterol interactions may influence the domain size distribution.

Adding O-lysoPC to eSM/DOPC/Chol = 1:1:1 membrane manifested only monotonic decrease of the domain size with an increase of the lysolipid concentration (Figure 3C). Concentrations of 1.0 and 2.0 mol% of O-lysoPC distinctly reduced the domain size to (130 ± 40) nm and (80 ± 30) nm, respectively. The further increase to 3.0 and 4.0 mol% of O-lysoPC kept the size of domains almost constant at around (70 ± 30) nm (Figure 3D).

While an average Δh value was around 1 nm for both lysolipids in eSM/DOPC/Chol = 1:1:1 membrane, we detected an increase of the Δh for 1 mol% of lysolipids (Figure 3). In general, increase of the raft height corresponds to the bigger line tension (García-Sáez et al., 2007). In contrast, we observed a strong decrease of the raft size in this case. Similar effect has been detected in (Bao et al., 2011) for the addition of ganglioside GM1 to eSM/DOPC/Chol = 2:2:1 membrane. Authors interpreted it as some artifact related to the fact that AFM measures relative height difference.

#### 3.1.3 DOG in eSM/DOPC/Chol = 2:2:1 membrane

**FIGURE 4:**
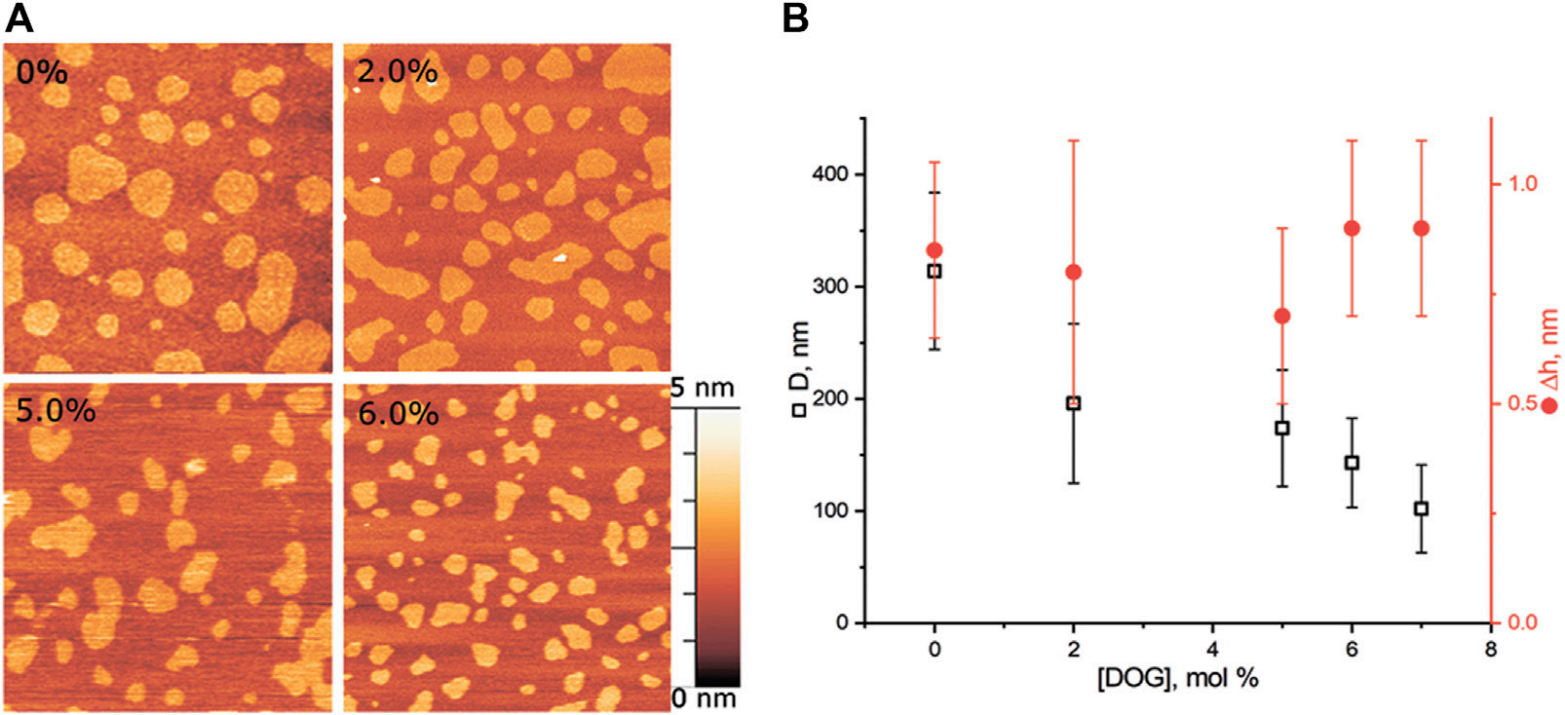
DOG in the eSM/DOPC/Chol = 2:2:1 membrane. **(A)** AFM images of the membrane. Size of each image is 3 μm × 3 μm. Amount of DOG in the membrane in mol% is indicated in each image. **(B)** Dependence of the average diameter of rafts (black) and their height above the surrounding membrane (red) on the molar fraction of DOG in the membrane.

We made a series of experiments for DOG, which induces negative spontaneous curvature in monolayers, i.e., the spontaneous curvature of the opposite sign as compared to lysolipids. Addition of DOG to the eSM/DOPC/Chol = 2:2:1 membrane gradually decreased the average raft size (Figure 4). The raft diameter was reduced twice at the concentration of 6 mol% of DOG, as compared to the DOG-free membrane (Figure 4B). Δh values were independent on DOG concentration, in a range from 0.5 to 1.1 nm.

#### 3.1.4 DOG in eSM/DOPC/Chol = 1:1:1 membrane

**FIGURE 5:**
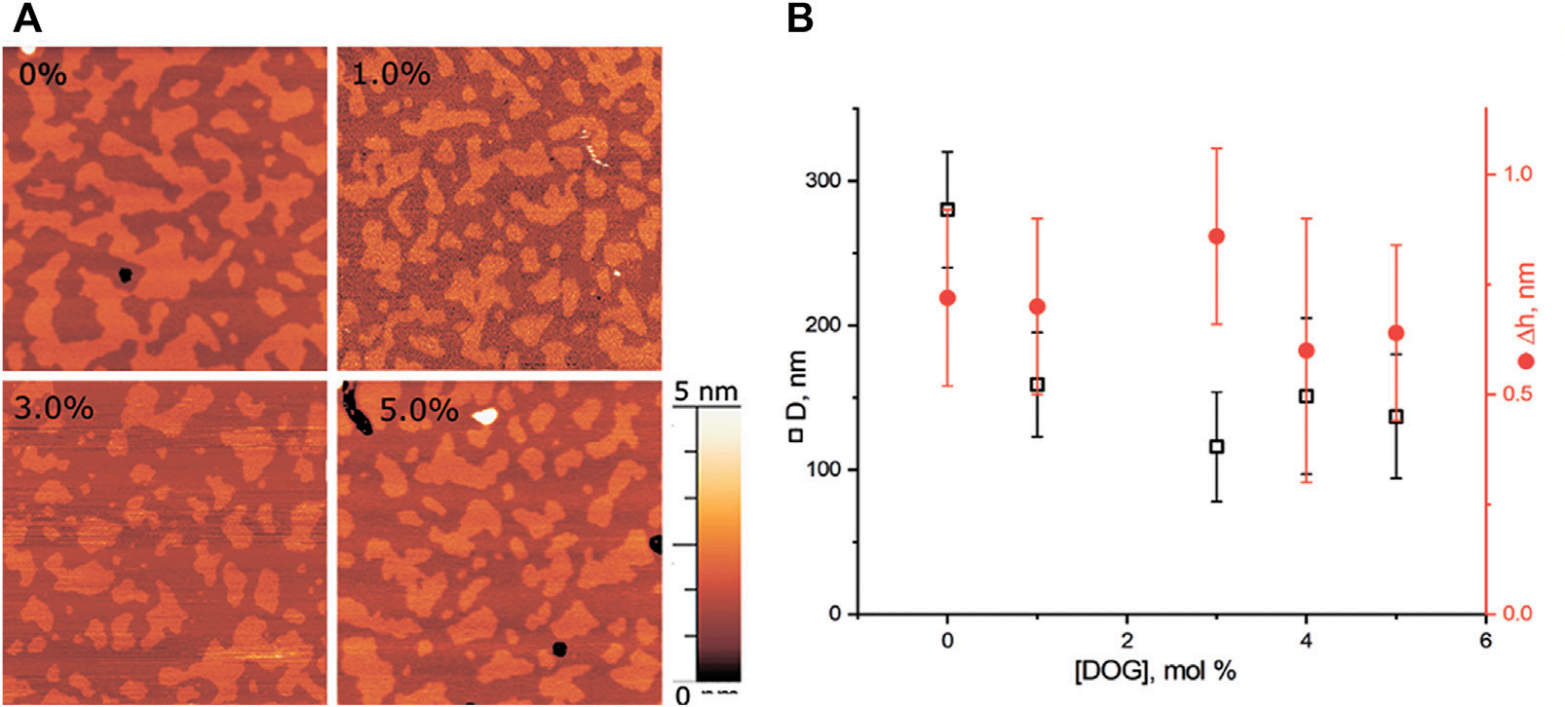
DOG in the eSM/DOPC/Chol = 1:1:1 membrane. **(A)** AFM images of the membrane. Size of each image is 3 μm × 3 μm. Amount of DOG in the membrane in mol% is indicated in each image. **(B)** Dependence of the average diameter of rafts (black) and their height above the surrounding membrane (red) on the molar fraction of DOG in the membrane.

Adding DOG to eSM/DOPC/Chol = 1:1:1 membrane, which has a higher cholesterol content, reduced the average size of rafts from (280 ± 70) nm to (160 ± 40) nm at the concentration of 2 mol% of DOG (Figure 5). For the higher mole fractions of DOG, the size of domains remained around this level (Figure 5B). Δh values were independent on DOG concentration, with a dispersion from 0.25 nm to 1.1 nm.

### 3.2 Theoretical analysis

To clarify the physicochemical mechanism of lysolipid and DOG influence on the line tension of the raft boundary, we studied the distribution of added species between the boundary region and the bulk membrane using a continuum elastic model (Galimzyanov et al., 2017). The energy of the raft boundary was assumed to be determined by deformations arising to compensate for the thickness mismatch between the thicker bilayer of the raft and thinner surrounding membrane. We calculated the energy of the raft boundary as a function of the lateral distribution of the added substance (lysolipids or DOG), which is shown to enrich mainly in a narrow stripe parallel to the boundary (Pinigin et al., 2020). A vicinity of the raft boundary is the most deformed region of the lipid bilayer, and line-active components enriching there allow partial relaxation of the elastic stress (Pinigin et al., 2020). We modeled added substances as components that can distribute both into the bulk phase and into the narrow stripe at the raft boundary region. The distribution coefficients were found by minimization of the total elastic energy of the system. The phase composition of lipid mixture was taken from the eSM/DOPC/Chol ternary phase diagram presented in the ref (Khadka et al., 2015), and summarized in Table 1.

**TABLE 1:** Phase compositions and spontaneous curvatures.

Component ratio (eSM/DOPC/Chol)	Liquid-ordered phase		Liquid-disordered phase	
	Composition	Spontaneous curvature	Composition	Spontaneous curvature
1:1:1	48% eSM	−0.15 nm−^1^	11% eSM	−0.14 nm−^1^
	5% DOPC		72% DOPC	
	47% Chol		17% Chol	
2:2:1	63% eSM	−0.08 nm−^1^	17% eSM	−0.10 nm−^1^
	2% DOPC		75% DOPC	
	35% Chol		8% Chol	

We calculated the dependence of the line tension of the raft boundary on overall lysolipid content in the membrane for two sets of parameters. The first set corresponded to the eSM:DOPC:Chol = 1:1:1 system: *B*_*o*_ = 30 *k*_*B*_ *T* (*k*_*B*_ *T* ∼ 4 × 10^−21^ J), *B*_*d*_ = 10 *k*_*B*_ *T*, *K*_*t*_ = 10 *k*_*B*_ *T*/nm^2^ = 40 mN/m, *h*_*d*_ = 1.3 nm, *h*_*o*_ = 1.8 nm, *J*_*d*_ = −0.14 nm^−1^, *J*_*o*_ = −0.15 nm^−1^ (Hamm and Kozlov, 2000; Baumgarrt et al., 2005; Risselada and Marrink, 2008; Perlmutter and Sachs, 2011; Kollmitzer et al., 2013) (Table 1). The second set of parameters was for the eSM:DOPC:Chol = 2:2:1 system, and differed only in the spontaneous curvatures of ordered and disordered phases: *J*_*d*_ = −0.1 nm^−1^, *J*_*o*_ = −0.08 nm^−1^ (Table 1). Although O-lysoPC and P-lysoPC lipids can differently distribute between L_o_ and L_d_ bulk phases, calculations demonstrated that this factor did not affect the dependence of the line tension on the concentration of the additives.

It was experimentally shown that lipid mixtures containing lysolipids and cholesterol can phase separate forming ordered domains, most probably, enriched by these two lipids (Karpunin et al., 2005). Lysolipids can form a hydrogen bond with cholesterol, while “common” two-tail glycerophospholipids cannot. This H-bond-mediated pairwise attraction of cholesterol and lysolipids is supposed to be supplemented by an energy gain upon a favorable packing of molecular species of opposite spontaneous curvature (negative of cholesterol and positive of lysolipids) in a nearly planar membrane. Even this “elastic” gain alone was shown to be sufficient to yield a phase separation in the DOPC:O-lysoPC:Chol mixture (Galimzyanov and Akimov, 2011) observed experimentally (Karpunin et al., 2005). Thus, here we assumed that lysolipid molecules formed heterodimers with cholesterol. The heterodimer was assumed to have a larger (more positive) spontaneous curvature than cholesterol does.

The spontaneous curvature of the heterodimer with P-lysoPС is taken equal to be *J*_*PPC*_ = −0.1 nm^−1^, for heterodimer with O-lysoPC—*J*_*OPC*_ = −0.2 nm^−1^. Spontaneous curvature of DOG was taken as *J*_*DOG*_ = −0.99 nm^−1^ (Szule et al., 2002).

**FIGURE 6:**
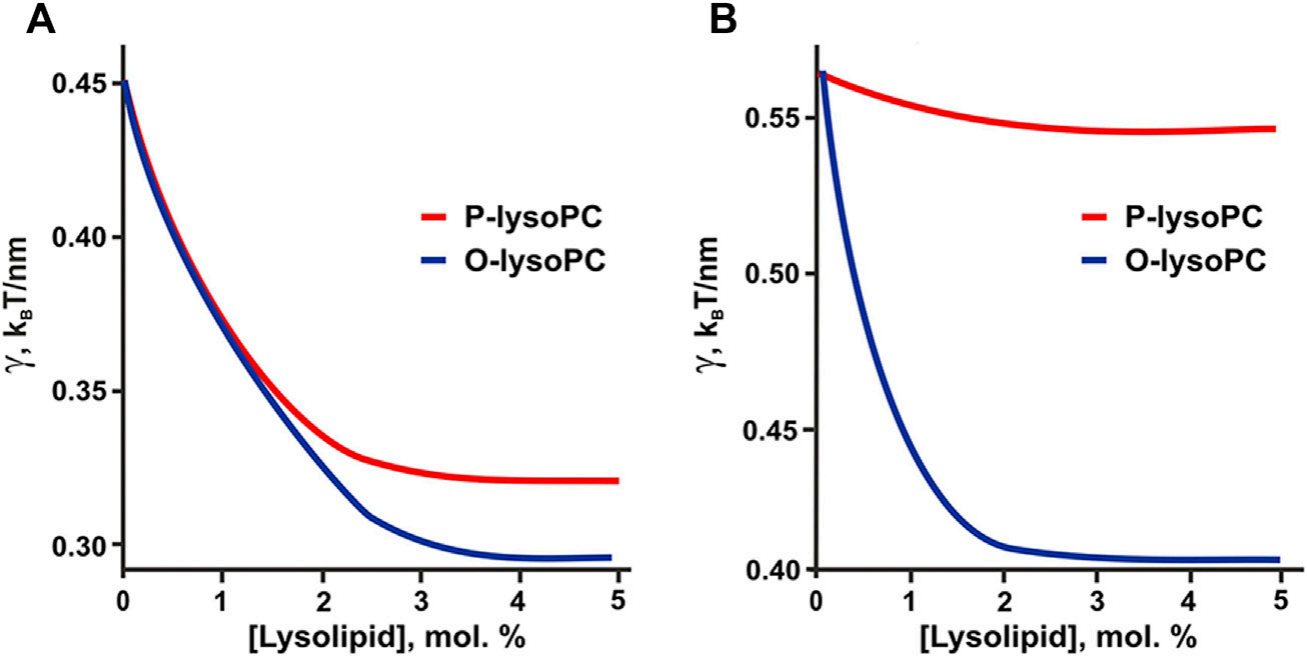
Calculated dependence of the line tension of the raft boundary on the total lysolipid content in the eSM:DOPC:Chol membrane. **(A)** eSM/DOPC/Chol = 1:1:1. **(B)** eSM/DOPC/Chol = 2:2:1.

Small fraction of lysolipids significantly decreased the line tension of the raft boundary due to the redistribution of the line-active component (i.e., lysolipid-cholesterol heterodimer) to the boundary region (Figure 6). Results of calculations correlate with AFM experiments. Weak dependence of raft boundary line tension on the P-lysoPC concentration in the 2:2:1 system (Figure 6B) is in line with the almost zero effect of this lipid found in experiments (Figure 2B). This behavior owes to the fact that the spontaneous curvature of P-lysoPC-cholesterol heterodimer almost equals to the average spontaneous curvatures of the L_o_ and L_d_ phases, thus the redistribution of this component cannot relax the stress at the boundary of L_o_ domains.

**FIGURE 7:**
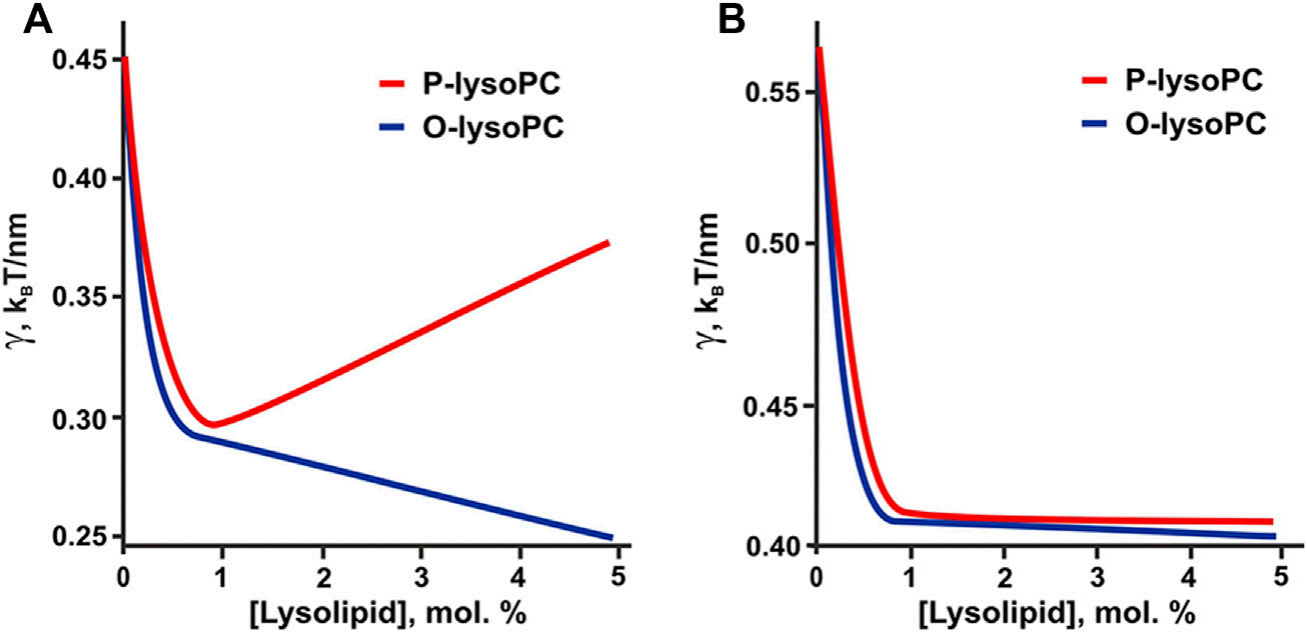
Calculated dependence of the line tension of the raft boundary on the total lysolipids content in the eSM:DOPC:Chol membrane for experimentally measured spontaneous curvatures of lysolipids: *J*_*OPC*_ = +0.26 nm^−1^, *J*_*PPC*_ = +0.15 nm^−1^. **(A)** eSM/DOPC/Chol = 1:1:1. **(B)** eSM/DOPC/Chol = 2:2:1.

The hypothesis of the formation of lysolipid-cholesterol heterodimers is indirectly confirmed by the results of our calculations. If we take the experimentally determined values of spontaneous curvatures of pure lysolipids: *J*_*OPC*_ = +0.26 nm^−1^, *J*_*PPC*_ = +0.15 nm^−1^ (Fuller and Rand, 2001), then in 1:1:1 system the line tension would be a non-monotonic function of P-lysoPC concentration (Figure 7A), similar to the case of addition of ganglioside to this system (Galimzyanov et al., 2017). This contradicts experimental results obtained here for P-lysoPC (Figure 3). In the 2:2:1 system the dependencies of the line tension (calculated without the assumption of heterodimer formation) on the concentrations of the P-lysoPC and O-lysoPC appeared to be almost identical (Figure 7B), which is also not in line with the experimental data (compare with Figures 2B,D).

**FIGURE 8:**
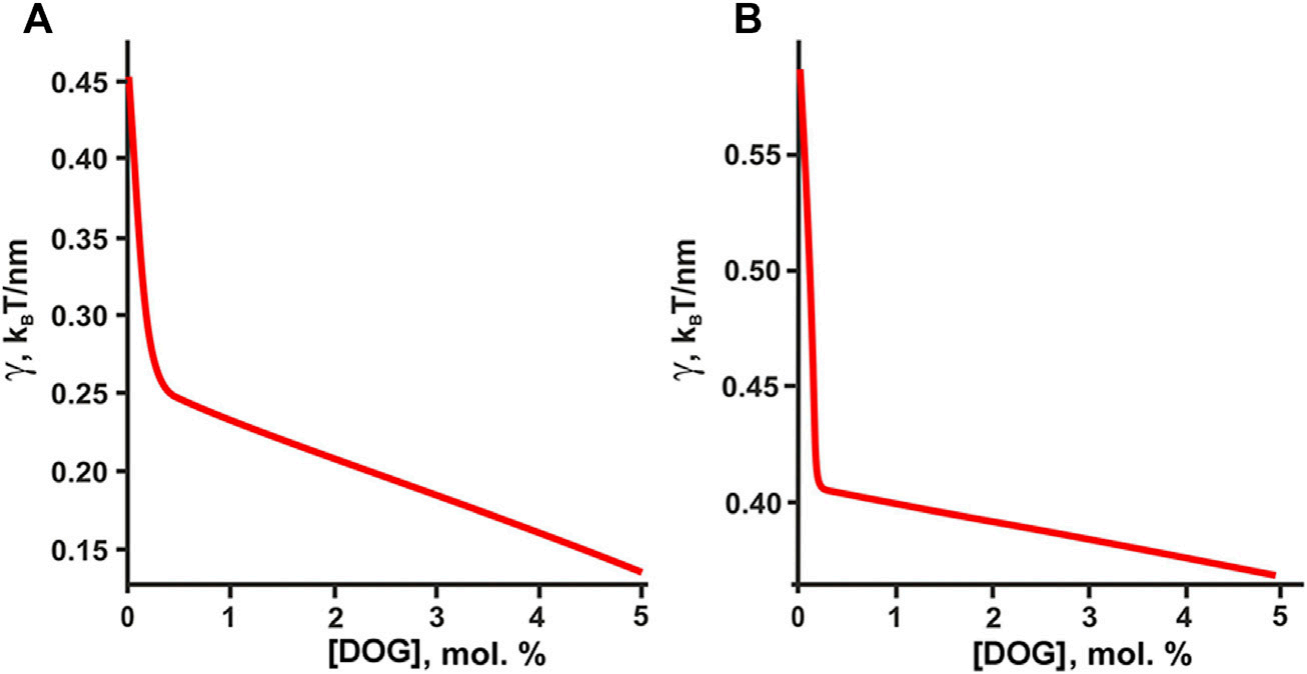
Calculated dependence of the line tension of the raft boundary on the total DOG content in the eSM:DOPC:Chol membrane. **(A)** eSM/DOPC/Chol = 1:1:1. **(B)** eSM/DOPC/Chol = 2:2:1.

Formally, DOG having unsaturated oleoyl tails should distribute predominantly into the L_d_ phase rich in DOPC. However, it was shown that pairwise interaction energies of molecular species decrease as the difference in their spontaneous curvature increases (Galimzyanov and Akimov, 2011). Due to extremely large spontaneous curvature, DOG molecule should strongly attract all molecular species in the system and thus should distribute almost equally between the coexisting L_o_/L_d_ phases, while the interactions related to chemical structure of lipid tails (saturated/unsaturated, etc.) should make minor contribution to the DOG lateral distribution between bulk phases. From this consideration, the addition of DOG is predicted to decrease the domain line tension (Figure 8), which correlates well with the reduction of the rafts size revealed in AFM experiments (Figures 4, 5).

## 4 Discussion

Lysolipid production in cellular membranes accompanies a lot of pathogenic processes, such as inflammations and some types of neurodegenerative diseases (Girotti and Kriska, 2004; Scholte et al., 2019). A high level of lysolipids may lead to damage of membranes by producing pores (Chernomordik et al., 1995). Molecular geometry of lysolipids suggests that they form a monolayer with positive spontaneous curvature, which favors the shape of the pore edge and disrupts the normal packing of lipid matrix of cellular membranes. However, substances possessing spontaneous curvature of the opposite sign, such as cholesterol, may compensate for these effects thus diminishing the negative effects of lysolipids on cell membrane integrity (Khandelia et al., 2014). Thus, one may conclude that the interplay between the levels of lysolipids and cholesterol in cells regulates the stability of their membranes.

Recently we have demonstrated that rafts in cellular membranes accumulate species with non-zero spontaneous curvature near their boundaries (Pinigin et al., 2020). Being enriched in a narrow stripe near the L_o_ domain boundary these substances exhibit so-called line activity changing the line tension of the domain boundary and, consequently, the equilibrium size distribution of the domains. Lipid rafts serve as platforms for many membrane proteins regulating their functionality (Simons and Ikonen, 1997; Deans et al., 2002; Parton and Richards, 2003; Salaün et al., 2004). Thus, change in the domain size may influence raft-related cellular processes. Good example of the molecule with the large positive spontaneous curvature is ganglioside GM1, for which we have demonstrated the significant effect of a very small amount of this glycolipid on the raft size distribution; the effect depends on the amount of cholesterol in the membrane (Galimzyanov et al., 2017). GM1 is an important regulatory lipid, and change in its level is associated with neurodegenerative disorders, especially AD. In the present study we further developed our previous model of line activity to consider the case of lysolipids. Within the same elastic approach (Pinigin et al., 2020) we theoretically predicted and experimentally proved that lysolipids may also serve as line-active substances in the membrane. To generalize our model we also considered DOG, which is the molecule characterized by the opposite, negative spontaneous curvature. Both our AFM experiments and theoretical calculations manifested the validity of the model for this type of molecules too. Similar to GM1, the effect of lysolipids on the size distribution of ordered domains was dictated by the cholesterol amount in the membrane. Cholesterol possesses the most negative spontaneous curvature of all the components used in our membrane systems (except that of DOG), thus, its content generally determines the overall spontaneous curvature of the phases and intermediate region between them (Figure 1). After saturating the stripe at the domain boundary, the species with non-zero spontaneous curvature have to distribute into the bulk phases. Formally, lysolipids utilized here should distribute in accordance with saturation of their hydrocarbon tails: P-lysoPC predominantly into the L_o_ phase, while O-lysoPC—predominantly into the L_d_ phase. However, there is increasing evidence that lysolipids and cholesterol can form a heterodimer; the mechanisms underlying the lateral distribution of heterodimers are not so straightforward. Formation of the heterodimers is in line with our results: calculations performed for elastic parameters of pure P-lysoPC and O-lysoPC yielded dependences of the line tension on lysolipid concentration inconsistent with the obtained experimental data, while calculations made under assumption of heterodimers formation led to consistent results. Moreover, the observed increase of the height difference between L_o_ and L_d_ phases for only 1 mol% of lysolipids in eSM:DOPC:Chol = 1:1:1 membrane (Figure 3) pointed at the fact that some changes in the inner structure of the raft might result in apparent changes in Δh. Our hypothesis is that around 1 mol% of lysolipids completely filled the transition region between the raft and surrounding membrane with lysolipid-cholesterol heterodimers. It resulted in an increase of the relative Δh. With increased amount of lysolipids in the membrane they started to distribute between the bulk L_o_/L_d_ phases thus decreasing Δh. However, the lateral size of the intermediate region of around 2 nm (Galimzyanov et al., 2015) prevented us from its clear imaging by AFM.

According to our theoretical model, any membrane component, the spontaneous curvature of which differs from that of the bulk phases, can manifest the line activity. Despite our model takes into account only “elastic” part of the free energy of the system based on the molecular shape, it provides good qualitative description of the line activity of membrane components. However, quantitative values of the line tension should be corrected by the chemical interactions of the membrane components. Recently, the line activity was demonstrated for GM1—the lipid with positive spontaneous curvature (Bao et al., 2011; Galimzyanov et al., 2017). Raft-forming lipid mixtures contain a substantial fraction of cholesterol (Simons and Ikonen, 1997; Heberle and Feigenson, 2011), the spontaneous curvature of which is highly negative and thus it determines the average spontaneous curvature of bulk phases. Here, using DOG, the spontaneous curvature of which is even more negative than that of cholesterol, we demonstrated for the first time (as far as we know) the possibility of line activity of membrane components with negative spontaneous curvature thus proving the general mechanism of the line activity.

## Data Availability

The raw data supporting the conclusion of this article will be made available by the authors, without undue reservation.
